# Visual acuity, amblyopia and refractive error in preterm children with and without retinopathy of prematurity – Results from the Gutenberg Prematurity Study Young (GPSY)

**DOI:** 10.1111/aos.17515

**Published:** 2025-05-28

**Authors:** Achim Fieß, Sandra Gißler, Alica Hartmann, Eva Mildenberger, Heike Elflein, Panagiotis Laspas, Christina Korb, Bernhard Stoffelns, Norbert Pfeiffer, Stephanie Grabitz, Alexander K. Schuster

**Affiliations:** ^1^ Department of Ophthalmology University Medical Center of the Johannes Gutenberg University Mainz Mainz Germany; ^2^ Division of Neonatology, Department of Pediatrics University Medical Center of the Johannes Gutenberg University Mainz Mainz Germany

**Keywords:** amblyopia, epidemiology, preterm birth, refractive error, ROP, visual acuity

## Abstract

**Purpose:**

This study aims to assess visual outcomes in children born preterm, stratified by gestational age, hypo‐ and hypertrophy, and the presence of retinopathy of prematurity (ROP) and its treatment.

**Methods:**

This is a prospective observational cohort study (*n* = 949, 1889 eyes) assessing visual acuity, amblyopia, refractive error, and lens opacifications in a large sample of children born preterm and full‐term aged 4–17 years. Covariates included gestational age, birth weight percentile, ROP status and treatment, maternal smoking, placental insufficiency, preeclampsia, breastfeeding, and perinatal adverse events with an adjustment for sex and age. Logistic regression analysis was conducted to evaluate associations.

**Results:**

Amblyopia prevalence was mainly associated with a gestational age ≤ 28 weeks (OR = 2.92, *p* = 0.03), placental insufficiency (OR = 3.84, *p* = 0.01), and ROP treatment (OR = 15.71, *p* ≤ 0.001). Distant corrected visual acuity (DCVA) in the better eye was significantly correlated with gestational age (*ρ* = −0.083; *p* = 0.01), birth weight (*ρ* = −0.096; *p* = 0.004), birth weight percentile (*ρ* = −0.064; *p* = 0.05), ROP (*ρ* = 0.13; *p* <0.001), ROP treatment (*ρ* = 0.21; *p* <0.001), and perinatal adverse events (*ρ* = 0.135; *p* <0.001). The spherical equivalent was associated only with ROP treatment [*β* = −2.91, *p* < 0.001]. Lens opacifications were significantly larger in the group treated for ROP (*p* ≤ 0.01).

**Conclusions:**

This study highlights that perinatal factors associated with prematurity affect visual acuity and refractive error as well as the development of amblyopia in children. Mainly, extreme prematurity ≤28 weeks as well as ROP and its treatment are the most important factors affecting visual development.

## 1 INTRODUCTION

Preterm birth is a critical issue that is associated with several ocular alterations from early childhood up to adulthood. One of the most serious conditions is retinopathy of prematurity (ROP), a retinal disorder that can lead to decreased visual function and even blindness if not properly managed (Kong et al., 2012). The increasing survival rates of extremely preterm infants have led to a growing prevalence of ROP worldwide (Blencowe et al., 2013, 2016). Prematurity, along with the occurrence and treatment of ROP, is linked to several visual impairments in childhood, including myopia, astigmatism, anisometropia, and strabismus (Darlow et al., 1997; Fieß, Kolb‐Keerl, Knuf, et al., 2017; Holmstrom et al., 1999; O'Connor et al., 2006; Schalij‐Delfos et al., 2000). These alterations significantly affect visual development in children, with potential long‐term impacts on visual acuity (Darlow et al., 2018; Pétursdóttir et al., 2021) and quality of life (Fieß, Greven, et al., 2022). Moreover, preterm children who have been treated for ROP (particularly with laser‐ and cryotherapy) often display thicker lenses, which may be linked to altered lens development, reduced accommodation, and angle‐closure glaucoma (Nagao & Chen, 2006; Saxena et al., 1993; Trigler et al., 2005). However, despite the importance of early visual development, there is a lack of comprehensive data on how prematurity and ROP affect refractive and lens development as well as visual function in children, particularly across different levels of prematurity. While research has predominantly focused on extremely preterm infants, the vast majority of preterm births occur in moderately preterm infants (gestational age (GA) ≥ 32 weeks) (Chawanpaiboon et al., 2019), who remain underrepresented in ophthalmological studies. This creates a gap in understanding how different degrees of prematurity, combined with ROP occurrence and treatment, influence visual function, the occurrence of amblyopia, ocular morphology, and refractive error in childhood. Low GA and birth weight (BW) are correlated closely, and therefore, the effects of low BW in correlation to GA indicated by BW percentile should be taken into account as a surrogate marker for the influence of hypo‐ or hypertrophy on ocular development. In current research, differentiation between low BW and prematurity often is blurred, which is why a structured approach, accounting for prematurity without ROP, ROP occurrence and treatment, as well as BW percentile, is highly necessary.

All of these factors may impact visual development, leading to the occurrence of strabismus or amblyopia due to refractive error, anisometropia, or opacification in the optical axis. Amblyopia is a condition that can only be treated sufficiently during younger childhood but may result in the absence of three‐dimensional vision and in decreased visual acuity in individuals up to adulthood.

Therefore, this study aims to assess the prevalence of amblyopia and the reasons for amblyopia in relation to prematurity and the effects of preterm birth on visual acuity, refractive error, and lens opacifications in children born preterm – stratified by GA and the presence of ROP – in comparison to children born full‐term with and without hyper‐ and hypotrophy. By focusing on children and adolescents, this study seeks to provide vital insights that may help shape future preventive and therapeutic strategies for improving the visual health of preterm infants, children, adolescents, and adults.

## 2 MATERIALS AND METHODS

### 2.1 Study population

The Gutenberg Prematurity Study Young (GPSY) is conducted at the University Medical Center of the Johannes Gutenberg University Mainz (UMCM) in Germany. This study focuses on a cohort of children and adolescents, aged 4–17, born either preterm or full‐term between 2003 and 2018, to explore the impact of prematurity and associated factors. The GPSY is designed as a retrospective cohort study with a prospective ophthalmologic assessment, extending to the Gutenberg Prematurity Eye Study (GPES) for adults (Fieß, Berger, et al., 2022; Fieß, Fauer, et al., 2022; Fieß, Gißler, Fauer, et al., 2022; Fieß, Gißler, Mildenberger, Urschitz, Fauer, et al., 2022a; Fieß, Gißler, Mildenberger, Urschitz, Zepp, et al., 2022b; Fieß, Grabitz, et al., 2022; Fieß, Greven, et al., 2022; Fieß, Hufschmidt‐Merizian, et al., 2022; Fieß, Nauen, et al., 2022; Fieß, Pfisterer, et al., 2022; Fieß, Schäffler, et al., 2022; Fieß, Schulze, et al., 2022; Fieß et al., 2019; Fieß, Zange, et al., 2024). Perinatal data were collected retrospectively by reviewing medical records from the UMCM, while the prospective phase involved comprehensive eye examinations.

Participants were selected using an algorithm that invited all individuals born preterm with a GA of ≤32 weeks and randomly selected one in three preterm individuals with a GA of 33–36 weeks. Control subjects were chosen by randomly selecting 8 individuals born full‐term (4 male, 4 female) per month of birth, with a BW between the 10th and 90th percentiles. Additionally, 30 participants born at term and severely small for GA (SGA), 30 participants born at term and moderately SGA, 30 individuals born at term and moderately large for GA (LGA), and 30 individuals born at term and severely LGA were recruited with stratification by sex and age. A detailed flow chart for eligibility can be found in Figure S1. The participants underwent examinations between 2022 and 2023 and provided additional information about their medical history through surveys.

Informed written consent was obtained from all participants and their legal guardians before enrolment. The GPSY adheres to Good Clinical Practice (GCP), Good Epidemiological Practice (GEP), and the ethical guidelines of the Declaration of Helsinki. The study protocol and documentation were approved by the local ethics committee of the Medical Chamber of Rhineland‐Palatinate, Germany (reference no. 2021‐15830; original approval: May 5, 2021; most recent update: January 19, 2022).

### 2.2 Assessment of pre‐, peri‐, and postnatal medical history

Medical records at the UMCM were examined for various data points, including GA (in weeks), BW (in kg), presence and stage of ROP, ROP treatment, placental insufficiency, preeclampsia, breastfeeding status, maternal smoking during pregnancy, hemolysis, elevated liver enzymes, and low platelet count (HELLP) and any perinatal adverse events, defined according to the German query for quality control of the neonatal clinics: occurrence of intraventricular haemorrhage (at least grade 3 or parenchymal haemorrhage) and/or the occurrence of necrotizing enterocolitis and/or moderate or severe bronchopulmonary dysplasia were summarized as adverse events (Institute for Quality Assurance and Transparency in Healthcare (IQTIG), 2023). BW percentiles were calculated based on the methodologies established by Voigt et al. (2006).

### 2.3 Categorization

Participants were divided into six groups based on GA and the presence of ROP. Group 1 included term‐born children with a GA at birth of 37 completed weeks or more. Group 2 comprised participants born preterm with a GA of 33 to 36 weeks who did not have ROP. Group 3 consisted of those with a GA of 29–32 weeks without ROP, while Group 4 included participants with a GA of 28 weeks or less, also without ROP. Group 5 was made up of participants with a GA of 32 weeks or less and non‐treated ROP, and Group 6 included those with treated ROP. If only one eye was affected by ROP, the unaffected non‐ROP eye was excluded from the analysis.

### 2.4 Ophthalmologic examination

The detailed ophthalmologic examination included testing of visual acuity without correction and distant corrected visual acuity (DCVA) with ARK 1 s (NIDEK; Oculus, Wetzlar, Germany). Additionally, the intraocular pressure was measured with a non‐contact tonometer (NT 2000; Nidek Co., Japan). A slit‐lamp examination of the anterior segment was performed, and a funduscopy was carried out. According to medical literature, visual acuity was converted from decimal to logMAR (Bach & Kommerell, 1998). Stereopsis was tested with the Lang‐II test. Strabismus was determined using the cover test. Participants underwent pharmacological cycloplegia using phenylephrine 5 mg/mL and tropicamide 5 mg/mL.

### 2.5 Refractive error, accommodation, and lens opacification

The spherical equivalent was calculated by summing the sphere value with half of the cylindrical value. Anisometropia was determined by calculating the absolute difference in spherical equivalents between the right and left eyes. Anisoastigmatism was assessed by measuring the absolute difference in cylindrical values between the two eyes. For the assessment of risk factors for amblyopia, cylindrical and spherical anisometropia were assessed if any of the differences were above 1 dioptre. Additionally, the occurrences of cataract and ptosis were evaluated with slit lamp examination, and a questionnaire also asked for the presence and acquirement of congenital and childhood cataracts. Opacifications of the optical media were evaluated by using retro‐illumination images taken by ARK 1s (NIDEK; Oculus, Wetzlar, Germany). The device used provides cataract indices that indicate the severity of the opacity, including the size of opacification within a central 3 mm zone (COI‐H), the proportion of opacification within this central zone as a percentage (COI‐A), and the opacity proportion throughout the entire periphery as a percentage (POI).

Each retro‐illuminated image was thoroughly reviewed, and any measurements with irregular alignment or incorrect pupil detection were excluded from the analysis.

### 2.6 Definition of amblyopia

Amblyopia was defined according to the definition in the Gutenberg Health Study (Elflein et al., 2015): Unilateral amblyopia was diagnosed when the best corrected vision in the worse eye was ≤0.63 with a two‐line difference between the eyes, or ≤0.5 without such a difference and presence or history of strabismus and/or anisometropia of ≥1.0 dioptre (spherical or cylindrical, affecting the weaker eye), and/or a history of visual deprivation. Importantly, no other ophthalmological abnormalities had to be present that could explain the limited vision. Bilateral amblyopia was defined as best corrected vision of ≤0.63 in both eyes and binocular hyperopia of ≥4.0 dioptres and/or bilateral astigmatism of ≥2.0 dioptres and/or bilateral myopia of ≥6.0 dioptres and/or a history of bilateral deprivation. As with unilateral amblyopia, no other ocular conditions should account for the reduced vision in bilateral cases. Additionally, in the comprehensive questionnaire, participants and their parents were asked if any amblyopia treatment (patching) had occurred.

To assess the main risk factor for amblyopia in the stratified groups, we grouped the reasons for amblyopia into the following order of most reasonable primary cause:

1. Deprivation by ptosis or cataract.
2. Strabismus.
3. Anisometropia ≥1 Dioptre.
4. Anisoastigmatism ≥1 Dioptre.
5. Refractive Error: Myopia ≥6.0 Dioptres or Hyperopia ≥4.0 Dioptres in both eyes.
6. Astigmatism ≥2 Dioptres in both eyes.

### 2.7 Covariates

The covariates included in the different models were potential factors that may influence our primary outcome measures. These covariates consisted of age (in years), sex (female), spherical equivalent (in dioptres), weeks of prematurity (40 weeks minus GA), BW (in kg), BW percentile, presence of ROP, ROP treatment, maternal smoking status, placental insufficiency, preeclampsia, breastfeeding status, and perinatal adverse events. Perinatal adverse events included the occurrence of intraventricular haemorrhage (at least grade 3 or parenchymal haemorrhage), necrotizing enterocolitis, and/or moderate or severe bronchopulmonary dysplasia, as defined by the German query for quality control in neonatal clinics (IQTIG, 2023).

### 2.8 Statistical analysis

Descriptive statistics were stratified by prematurity group. Absolute and relative frequencies were calculated for dichotomous parameters, and mean and standard deviation were calculated for approximately normally distributed variables (otherwise median and interquartile range). Associations between amblyopia and prematurity‐related factors were assessed by logistic regression analysis. In the case of continuous, not normally distributed variables (astigmatism, anisometropia, anisoastigmatism), quantile regression was applied, and only right eyes were included apart from the left eyes of participants whose eyes were both designated to different groups in the case of ROP. In this case, the eye in the higher group was included. Association analyses of spherical equivalent were conducted using linear regression with generalized estimating equations to account for both eyes.

For amblyopia, spherical equivalent (dioptre), astigmatism (dioptre), anisometropia (dioptre) and anisoastigmatism (dioptre), univariable analyses were conducted investigating the relationship with GA (weeks), BW (kg), BW percentile, ROP (yes), ROP treatment (yes), placental insufficiency (yes), preeclampsia (yes), maternal smoking (yes), perinatal adverse events (yes), and breastfeeding (yes). In multivariable regression model #1, all univariable associated parameters were included except BW, ROP occurrence, and ROP treatment due to its high correlation with GA. The associated parameters from model #1 and postnatal ROP occurrence and ROP treatment (if associated in univariable analyses) were included in a second model. All analyses were adjusted for sex (female) and age (years).

As this is an explorative study, a significance level was not defined and no adjustment for multiple testing was conducted. Thus, *p*‐values are reported only for descriptive purposes and should be interpreted with caution (Greenland et al., 2016). Calculations were performed using R (R Core Team 2021; R version 4.1.2 (2021‐11‐01)).

## 3 RESULTS

### 3.1 Study participants' characteristics

In the present study, 1889 eyes of 949 preterm and full‐term participants were included (mean age = 11.2 years, 495 female subjects). Overall, 760 eyes of 380 participants with a GA ≥ 37 weeks (group 1, control group), 484 eyes of 242 participants with a GA between 33 and 36 weeks without ROP (group 2), 340 eyes of 170 participants with a GA between 29 and 32 weeks without ROP (group 3), 156 eyes of 78 participants with a GA ≤ 28 weeks without ROP (group 4), 114 eyes of 60 participants with a GA between 24 and 32 weeks with ROP without treatment (group 5), and 35 eyes of 19 participants with a GA between 24 and 32 weeks and with postnatal treatment for ROP (group 6) were assessed. The group characteristics are displayed in Table S1.

### 3.2 Ocular parameters

Descriptive statistics for ocular parameters (visual acuity, stereopsis, amblyopia, and intraocular pressure) are displayed in Table 1, with reasons for amblyopia ordered by the most reasonable cause, while refractive error, astigmatism, anisoastigmatism, anisometropia, and lens opacifications are displayed in Table 2. None of the participants had ptosis.

**TABLE 1:** Parameters of the GPSY sample for each Study Group regarding visual acuity, stereopsis, amblyopia and intraocular pressure.

	Group 1, GA ≥ 37 wks	Group 2, GA 33–36 wks, No ROP	Group 3, GA 29–32 wks, No ROP	Group 4, GA ≤ 28 wks, No ROP	Group 5, GA ≤ 32 wks, ROP without treatment	Group 6, GA ≤ 32 wks, ROP with treatment
Visual acuity						
Participants visual acuity (*n*)	375	241	166	74	54	14
Eyes visual acuity (eyes)	749	481	312	147	103	26
DCVA (LogMAR) (OD) (median [IQR])	0.00 [0.00, 0.10]	0.00 [0.00, 0.10]	0.00 [0.00, 0.10]	0.00 [0.00, 0.20]	0.00 [0.00, 0.20]	0.20 [0.20, 0.30]
DCVA (LogMAR) (OS) (median [IQR])	0.00 [0.00, 0.10]	0.10 [0.00, 0.20]	0.03 [0.00, 0.10]	0.00 [0.00, 0.20]	0.05 [0.00, 0.16]	0.44 [0.10, 1.02]
Visual acuity better eye (logMAR) (median [IQR])	0.00 [0.00, 0.00]	0.00 [0.00, 0.10]	0.00 [0.00, 0.03]	0.00 [0.00, 0.10]	0.00 [0.00, 0.15]	0.20 [0.10, 0.65]
Visual acuity worse eye (logMAR) (median [IQR])	0.00 [0.00, 0.10]	0.10 [0.00, 0.20]	0.00 [0.00, 0.10]	0.10 [0.00, 0.20]	0.00 [0.00, 0.20]	0.30 [0.20, 0.49]
Visual acuity better eye (Decimal)						
<20/20 (Decimal) (*n* (%))	84 (22.4)	71 (29.5)	42 (25.3)	22 (29.7)	17 (31.5)	13 (92.9)
<20/40 (Decimal) (*n* (%))	10 (2.7)	1 (0.4)	2 (1.2)	3 (4.1)	3 (5.6)	3 (21.4)
<20/60 (Decimal) (*n* (%))	0 (0.0)	0 (0.0)	0 (0.0)	0 (0.0)	1 (1.9)	3 (21.4)
<20/200 (Decimal) (*n* (%))	0 (0.0)	0 (0.0)	0 (0.0)	0 (0.0)	0 (0.0)	2 (14.3)
<20/400 (Decimal) (*n* (%))	0 (0.0)	0 (0.0)	0 (0.0)	0 (0.0)	0 (0.0)	0 (0.0)
Stereopsis and amblyopia						
No stereopsis (Lang II test) (*n* (%))	8 (2.1)	6 (2.5)	6 (3.6)	6 (8.1)	2 (3.7)	9 (64.3)
Strabismus (yes) (*n* (%))	22 (5.9)	25 (10.4)	19 (11.4)	15 (20.3)	9 (16.7)	10 (71.4)
History of patching treatment (*n* (%))	14 (3.7)	14 (5.8)	7 (4.2)	15 (20.3)	3 (5.6)	11 (78.6)
Unilateral amblyopia at examination (*n* (%))	8 (2.1)	12 (5.0)	7 (4.2)	7 (9.5)	3 (5.6)	4 (28.6)
Bilateral amblyopia at examination (*n* (%))	3 (0.8)	0 (0.0)	1 (0.6)	0 (0.0)	0 (0.0)	4 (28.6)
Any amblyopia at examination (*n* (%))	11 (2.9)	12 (5.0)	8 (4.8)	7 (9.5)	3 (5.6)	8 (57.1)
Reason for amblyopia (*n* of any amblyopia)						
Cataract	0 (0.0)	1 (0.4)	0 (0.0)	1 (1.4)	1 (1.9)	1 (7.1)
Strabismus	5 (1.3)	10 (4.1)	4 (2.4)	5 (6.8)	2 (3.7)	6 (42.9)
Anisometropia ≥ 1 D	4 (1.1)	1 (0.4)	3 (1.8)	1 (1.4)	0 (0.0)	0 (0.0)
Anisoastigmatism ≥ 1 D	1 (0.03)	0 (0.0)	0 (0.0)	0 (0.0)	0 (0.0)	1 (7.1)
Myopia ≥ 6 D/Hyperopia ≥ 4 D in both eyes	0 (0.0)	0 (0.0)	1 (0.6)	0 (0.0)	0 (0.0)	0 (0.0)
High astigmatism ≥2 D	1 (0.3)	0 (0.0)	1 (0.6)	0 (0.0)	0 (0.0)	0 (0.0)
Intraocular pressure						
Measurement IOP (eyes OD/OS)	349/347	232/223	151/149	66/63	43/41	9/9
Intraocular pressure (mmHg) (OD) (mean (SD))	16.85 (3.02)	16.37 (2.72)	16.31 (3.10)	18.19 (3.88)	17.24 (4.23)	16.97 (3.62)
Intraocular pressure (mmHg) (OS) (mean (SD))	16.36 (2.93)	15.78 (2.81)	15.58 (3.02)	17.08 (3.38)	16.67 (4.88)	17.34 (4.32)

Abbreviations: DCVA, distant‐corrected visual acuity; GA, gestational age; IQR, interquartile range; mmHg, millimetre mercury; *n*, number of eyes; OD, right eye; OS, left eye; ROP, retinopathy of prematurity; SD, standard deviation; wks, weeks.

**TABLE 2:** Parameters of the GPSY sample for each Study Group regarding refractive error and lens opacifications.

	Group 1, GA ≥ 37 wks	Group 2, GA 33–36 wks, No ROP	Group 3, GA 29–32 wks, No ROP	Group 4, GA ≤ 28 wks, No ROP	Group 5, GA ≤ 32 wks, ROP without treatment	Group 6, GA ≤ 32 wks, ROP with treatment
Refractive error						
Participants refractive error (*n*)	376	239	164	73	55	14
Eyes refractive error (eyes)	750	477	328	145	105	27
Wearing glasses (yes) (*n* (%))	90 (23.9)	65 (27.2)	42 (25.6)	28 (38.4)	17 (30.9)	13 (92.9)
Spherical equivalent (D) (OD) (mean (SD))	0.24 (1.66)	0.30 (1.46)	0.40 (1.61)	0.21 (2.01)	0.39 (2.02)	−2.45 (4.67)
Spherical equivalent (D) (OS) (mean (SD))	0.25 (1.70)	0.34 (1.53)	0.48 (1.73)	0.36 (1.91)	0.29 (2.43)	−2.52 (4.89)
SE < −6 dioptre (eyes (%))	4 (0.5)	3 (0.6)	4 (1.2)	2 (1.4)	4 (3.8)	2 (7.4)
SE −6 to < −3 dioptre (eyes (%))	20 (2.7)	13 (2.7)	4 (1.2)	6 (4.1)	3 (2.9)	6 (22.2)
SE −3 to < −0.5 dioptre (eyes (%))	98 (13.1)	63 (13.2)	31 (9.5)	11 (7.6)	4 (3.8)	9 (33.3)
SE −0.5 to <0.5 dioptre (eyes (%))	299 (39.9)	171 (35.8)	119 (36.3)	57 (39.3)	43 (41.0)	2 (7.4)
SE 0.5 to <3.0 dioptre (eyes (%))	302 (40.3)	214 (44.9)	157 (47.9)	62 (42.8)	46 (43.8)	7 (25.9)
SE 3.0 to <6.0 dioptre (eyes (%))	24 (3.2)	13 (2.7)	13 (4.0)	7 (4.8)	3 (2.9)	1 (3.7)
SE ≥ 6.0 dioptre (eyes (%))	3 (0.4)	0 (0.0)	0 (0.0)	0 (0.0)	2 (1.9)	0 (0.0)
Anisometropia (Dioptres) (median [IQR])	0.22 [0.10, 0.38]	0.23 [0.10, 0.44]	0.23 [0.12, 0.48]	0.32 [0.11, 0.75]	0.38 [0.17, 0.56]	0.94 [0.42, 1.65]
Anisometropia (spherical)						
<0.5 dioptre (*n* (%))	306 (81.4)	183 (76.6)	125 (76.2)	46 (63.0)	33 (60.0)	5 (35.7)
0.5 to <1.0 dioptre (*n* (%))	42 (11.2)	40 (16.7)	26 (15.9)	14 (19.2)	13 (23.6)	2 (14.3)
1.0 to <1.5 dioptre (*n* (%))	15 (4.0)	6 (2.5)	7 (4.3)	6 (8.2)	1 (1.8)	3 (21.4)
1.5 to <2.0 dioptre (*n* (%))	4 (1.1)	3 (1.3)	3 (1.8)	2 (2.7)	1 (1.8)	1 (7.1)
2.0 to <2.5 dioptre (*n* (%))	2 (0.5)	4 (1.7)	1 (0.6)	3 (4.1)	1 (1.8)	1 (7.1)
2.5 to <3.0 dioptre (*n* (%))	2 (0.5)	1 (0.4)	1 (0.6)	0 (0.0)	0 (0.0)	0 (0.0)
Astigmatism (dioptre) OD (median [IQR])	−0.32 [−0.50, −0.20]	−0.36 [−0.64, −0.22]	−0.30 [−0.50, −0.20]	−0.41 [−0.65, −0.25]	−0.49 [−0.70, −0.25]	−1.96 [−2.45, −1.63]
Astigmatism (dioptre) OS (median [IQR])	−0.36 [−0.55, −0.23]	−0.35 [−0.65, −0.25]	−0.36 [−0.57, −0.22]	−0.35 [−0.94, −0.20]	−0.36 [−0.69, −0.25]	−1.61 [−2.50, −1.00]
Astigmatism categorical						
<0.5 dioptre (eyes (%))	507 (67.6)	312 (65.4)	224 (68.3)	82 (56.6)	58 (55.2)	0 (0.0)
0.5 to <1.0 dioptre (eyes (%))	188 (25.1)	106 (22.2)	71 (21.6)	35 (24.1)	35 (33.3)	5 (18.5)
1.0 to <1.5 dioptre (eyes (%))	32 (4.3)	29 (6.1)	18 (5.5)	11 (7.6)	5 (4.8)	3 (11.1)
1.5 to <2 dioptre (eyes (%))	9 (1.2)	8 (1.7)	6 (1.8)	10 (6.9)	2 (1.9)	7 (25.9)
2 to <4.0 dioptre (eyes (%))	12 (1.6)	22 (4.6)	5 (1.5)	6 (4.1)	5 (4.8)	11 (40.7)
≥4.0 dioptre (eyes (%))	2 (0.3)	0 (0.0)	4 (1.2)	1 (0.7)	0 (0.0)	0 (0.0)
Anisoastigmatism (dioptre) (median [IQR])	0.14 [0.06, 0.25]	0.14 [0.04, 0.25]	0.14 [0.05, 0.25]	0.16 [0.04, 0.37]	0.16 [0.05, 0.32]	0.78 [0.50, 1.54]
Anisoastigmatism (*n*)						
<0.5 dioptre (*n* (%))	344 (91.5)	213 (89.1)	147 (89.6)	60 (82.2)	45 (81.8)	3 (21.4)
0.5 to <1.0 dioptre (*n* (%))	21 (5.6)	15 (6.3)	15 (9.1)	9 (12.3)	2 (3.6)	5 (35.7)
1.0 to <1.5 dioptre (*n* (%))	6 (1.6)	6 (2.5)	1 (0.6)	1 (1.4)	2 (3.6)	2 (14.3)
1.5 to <2.0 dioptre (*n* (%))	1 (0.3)	2 (0.8)	0 (0.0)	1 (1.4)	1 (1.8)	2 (14.3)
2.0 to <2.5 dioptre (*n* (%))	0 (0.0)	1 (0.4)	0 (0.0)	0 (0.0)	1 (1.8)	1 (7.1)
2.5 to <3.0 dioptre (*n* (%))	0 (0.0)	1 (0.4)	0 (0.0)	0 (0.0)	0 (0.0)	0 (0.0)
Lens opacification						
Measurement lens opacification (eyes)	584	378	255	101	67	14
Opacification size within the 3 mm central zone (median [IQR])	0.00 [0.00, 0.00]	0.00 [0.00, 0.00]	0.00 [0.00, 0.00]	0.00 [0.00, 0.00]	0.00 [0.00, 0.00]	0.00 [0.00, 0.38]
Opacification proportion within the 3 mm central zone (median [IQR])	0.00 [0.00, 0.00]	0.00 [0.00, 0.00]	0.00 [0.00, 0.00]	0.00 [0.00, 0.00]	0.00 [0.00, 0.00]	0.00 [0.00, 6.00]
Opacity proportion within the entire periphery (median [IQR])	2.00 [0.00, 9.00]	3.00 [0.00, 11.00]	3.0 [0.00, 13.00]	1.00 [0.00, 8.00]	3.00 [0.00, 12.50]	7.00 [3.00, 13.00]

Abbreviations: GA, gestational age; IQR, interquartile range; mm, millimetre; *n*, number of eyes; OD, right eye; OS, left eye; ROP, retinopathy of prematurity; SD, standard deviation; wks, weeks.

**FIGURE 1:**
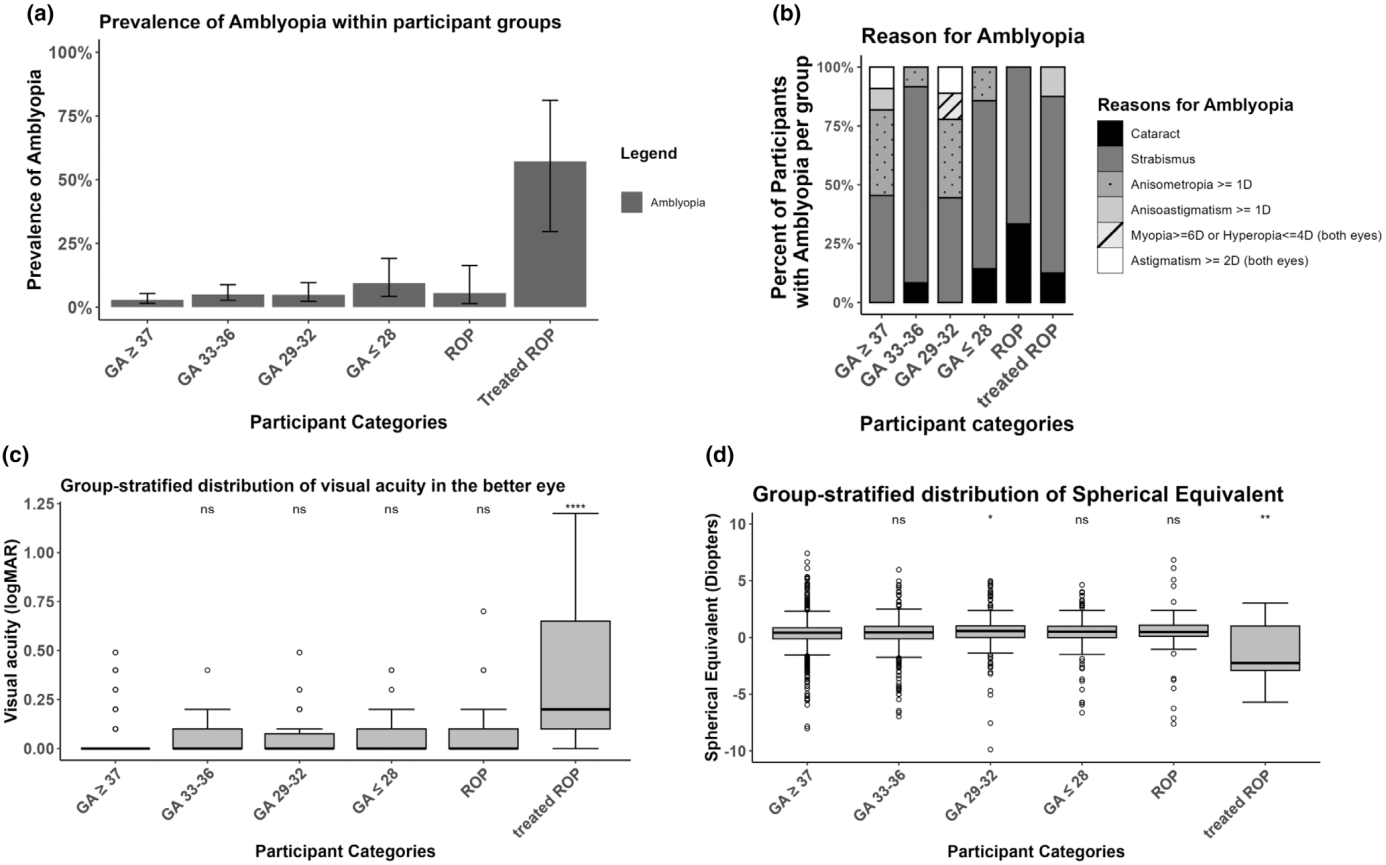
Graphical display of the reasons for amblyopia, refractive error and visual function. GA – gestational age in weeks, ROP – retinopathy of prematurity. Group‐stratified distributions of (a) prevalence of amblyopia, (b) reasons for amblyopia, (c) visual acuity, (d) spherical equivalent, comparisons against term‐born group (GA ≥37): ns, *p* > 0.05; *p ≤ 0.05; **p ≤ 0.01; ****p ≤ 0.0001, whiskers in a): 95% Confidence‐Interval.

Prevalence estimates and the distribution of reasons for amblyopia as well as visual acuity and refractive error are shown in Figure 1. Distributions of astigmatism and anisoastigmatism as well as strabismus and astigmatism are displayed in Figure S2.

### 3.3 Visual acuity in the better eye

Descriptively, the group treated for ROP had lower DCVA compared with controls (*p* ≤ 0.0001). Spearman correlation analysis revealed a univariable association of DCVA in the better eye with GA (*ρ* = −0.083; *p* = 0.01), BW (*ρ* = −0.096; *p* = 0.004), BW percentile (*ρ* = −0.064; *p* = 0.05), ROP (*ρ* = 0.13; *p* <0.001), ROP‐treatment (*ρ* = 0.21; *p*  <0.001) and perinatal adverse events (*ρ* = 0.135; *p* <0.001).

### 3.4 Amblyopia

In descriptive analyses, 11 children in the full‐term group (2.9%), 12 children (5.0%) in the GA group 33–36 weeks, 8 children (4.8%) in the GA group 29–32 weeks, 7 children (9.5%) in the GA group ≤28 weeks, 3 children (5.6%) in the untreated ROP group, and 8 children (57.1%) in the treated ROP group had amblyopia. Unilateral amblyopia was present in all groups, while bilateral amblyopia was only present in three full‐term children (0.8%), one child born with a GA of 29–32 weeks (0.6%) and four children with treated ROP (28.6%). The most prevalent reasons for amblyopia were mainly strabismus and anisometropia (≥1 dioptre).

GA as a continuous variable was associated with amblyopia in univariable and multivariable models (see Table S2), we decided to do a sensitivity analysis with grouped GA (see Table 3). In multivariable model 2, the odds of overall amblyopia increased significantly for the children with a GA of ≤28 weeks, compared with the ones born at term [OR = 2.92 (95% CI: 1.04; 7.77) *p* = 0.03], while moderate and late prematurity did not affect the prevalence of amblyopia significantly. Further, placental insufficiency more than tripled the odds of amblyopia [OR = 3.84 (95% CI: 1.25; 10.32), *p* = 0.01] and odds for amblyopia with ROP with treatment were 15 times higher than without [OR = 15.71 (95% CI: 3.61; 86.95), *p* <0.001], while ROP itself did not significantly alter the odds for amblyopia (see Table 3).

**TABLE 3:** Association analyses of ocular parameters.

Logistic regression	Univariable	*p*	Model 1	*p*	Model 2	*p*
	OR [95%‐CI]		OR [95%‐CI]		OR [95%‐CI]	
Amblyopia						
Gestational age (categorical)						
≥37 weeks	Ref.	—	Ref.	—	Ref.	—
33–36 weeks	1.75 [0.75; 4.11]	0.19	1.74 [0.74; 4.10]	0.20	1.62 [0.69; 3.83]	0.26
29–32 weeks	1.49 [0.57; 3.77]	0.40	1.45 [0.54; 3.71]	0.45	1.40 [0.53; 3.57]	0.48
≤28 weeks	4.64 [2.16; 10.43]	<0.001	2.56 [0.88; 7.09]	0.08	2.92 [1.04; 7.77]	0.03
Birth weight (kg)	0.45 [0.31;0.64]	<0.001	*	*	*	*
Birth weight percentile	0.99 [0.98;1.00]	0.11	—	—	—	—
ROP (yes)	4.85 [2.22;9.99]	<0.001	—	—	0.53 [0.11;1.97]	0.38
ROP treatment (yes)	24.64 [8.67;69.06]	<0.001	—	—	15.71 [3.61; 86.95]	<0.001
Perinatal adverse events (yes)	6.10 [2.89;12.35]	<0.001	2.30 [0.85; 6.38]	0.10	—	—
Smoking during pregnancy (yes)	4.48 [1.72;10.32]	<0.001	2.25 [0.78; 5.60]	0.10	—	—
Preeclampsia (yes)	2.40 [0.94;5.38]	0.05	0.51 [0.14; 1.41]	0.24	—	—
Breastfeeding (yes)	0.58 [0.30;1.16]	0.11	—	—	—	—
Placental insufficiency (yes)	3.88 [1.09;10.88]	0.02	3.52 [1.16; 9.50]	0.02	3.84 [1.25; 10.32]	0.01

Linear regression (GEE)	*β* [95%‐CI]	*p*	*β* [95%‐CI]	*p*	*β* [95%‐CI]	*p*
Spherical equivalent (Dioptres)						
Weeks of prematurity (40 weeks – GA)	−0.01 [−0.03;0.01]	0.40	—	—	—	—
Birth weight (kg)	0.02 [−0.06;0.11]	0.60	*	*	*	*
Birth weight percentile	0.001 [−0.002;0.004]	0.43	—	—	—	—
ROP (yes)	−0.47 [−1.00;0.06]	0.08	—	—	—	—
ROP treatment (yes)	−2.91 [−4.60; −1.21]	<0.001	—	—	—	—
Perinatal adverse events (yes)	−0.37 [−0.87;0.12]	0.14	—	—	—	—
Smoking during pregnancy (yes)	0.17 [−0.26;0.60]	0.44	—	—	—	—
Preeclampsia (yes)	−0.13 [−0.46;0.19]	0.42	—	—	—	—
Breastfeeding (yes)	0.15 [−0.06;0.35]	0.16	—	—	—	—
Placental insufficiency (yes)	0.07 [−0.50;0.65]	0.80	—	—	—	—

Quantile regression	*β*(τ50) [95%‐CI]	*p*	*β*(τ50) [95%‐CI]	*p*	*β*(τ50) [95%‐CI]	*p*
Anisometropia (Dioptres)						
Gestational age (categorical)						
≥37 weeks	Ref.	—	Ref.	—	Ref.	—
33–36 weeks	0.02 [−0.006; 0.06]	0.32	0.02 [−0.01; 0.05]	0.32	0.02 [−0.02;0.05]	0.46
29–32 weeks	0.02 [−0.03; 0.06]	0.38	0.01 [−0.03; 0.06]	0.65	0.009 [−0.04; 0.06]	0.76
≤28 weeks	1.81 [0.11;0.23]	<0.001	0.17 [0.08; 0.23]	<0.001	0.13 [0.02; 0.21]	0.02
Birth weight (kg)	−0.03 [−0.06; −0.01]	0.01	*	*	*	*
Birth weight percentile	−0.00 [−0.00;0.00]	0.62	—	—	—	—
ROP (yes)	0.19 [0.12;0.26]	<0.001	—	—	0.07 [0.01; 0.20]	0.23
ROP treatment (yes)	0.64 [0.20;1.51]	0.05	—	—	—	—
Perinatal adverse events (yes)	0.17 [0.05;0.26]	0.009	0.02 [−0.05; 0.16]	0.74	—	—
Smoking during pregnancy (yes)	0.00 [−0.07;0.05]	0.99	—	—	—	—
Preeclampsia (yes)	0.04 [0.02;0.17]	0.52	—	—	—	—
Breastfeeding (yes)	−0.005 [−0.05;0.03]	0.79	—	—	—	—
Placental insufficiency (yes)	−0.001 [−0.09;0.17]	0.99	—	—	—	—

*Note*: Univariable – adjusted for sex and age, Model 1: Multivariable model with inclusion of univariable associated parameters with adjustment for age and sex. Model 2: Multivariable model with inclusion of associated parameters of model 1 and additional inclusion of ROP occurrence/ROP treatment. Because of a high collinearity with gestational age the parameter birth weight was not included in the multivariable models. * removed from the analysis due to high collinearity.

Abbreviations: CI, confidence interval; OR, odds ratio.

### 3.5 Spherical equivalent

Spherical equivalent was significantly more myopic in individuals with ROP treatment compared with controls (*p* ≤ 0.01) (see Figure 1d). In univariable analyses, only ROP treatment was significantly associated with spherical equivalent [*β* = −2.91 (95% CI: −4.60; −1.21), *p* < 0.001] (see Table 3).

### 3.6 Anisometropia

In descriptive analysis, anisometropia was significantly higher in the group with GA ≤28 weeks (*p* ≤ 0.05), the group with ROP without treatment (*p* ≤ 0.001) and the group with ROP treatment (*p* ≤ 0.0001) compared with the full‐term group. In an analysis with GA as a continuous variable, GA was associated with anisometropia in univariable models as well as model 1 (see Table S2). When analysing GA as a categorical variable, anisometropia was only significantly associated with a GA ≤28 weeks, both in model 1 [*β*(τ50) = 0.17 (95% CI: 0.08; 0.23), *p* = <0.001] and 2 [*β*(τ50) = 0.13 (95% CI: 0.02; 0.21), *p* = 0.02].

### 3.7 Lens opacifications

In descriptive analyses, lens opacifications were significantly larger in the group treated for ROP compared with controls (*p* ≤ 0.01), while no other group showed a significant difference compared with controls.

## 4 DISCUSSION

This study provides a detailed assessment of perinatal parameters and their influence on ocular function and refractive error in childhood. Amblyopia prevalence was mainly influenced by extreme prematurity (GA ≤ 28 weeks), ROP treatment, and placental insufficiency. Refractive errors also showed several associations with perinatal parameters. Birth weight percentile was not associated with amblyopia, spherical equivalent, or anisometropia in our univariable models, for which we conclude that rather extreme prematurity and ROP, as well as ROP treatment, play a role in the development of amblyopia rather than perinatal hypo‐ or hypertrophy as a surrogate marker for fetal nutrition. Our findings emphasize the necessity for comprehensive ophthalmologic follow‐ups to facilitate early intervention for children with preterm birth and ROP history, as well as placental insufficiency, addressing the potential long‐term consequences on vision.

Our results imply a main effect of strabismus and anisometropia on the development of amblyopia, which has been described in previous studies (Pai et al., 2012). Both strabismus and anisometropia have been found to be related to prematurity (Fieß, Dautzenberg, et al., 2024; Holmstrom et al., 1999; Larsson & Holmstrom, 2006). According to the descriptive distribution of strabismus and anisometropia as causes for amblyopia in our cohort, one could speculate that strabismus is the major cause of amblyopia. Even in children that were not diagnosed with strabismus and therefore fell into the later categories, microstrabismus, which is not always uncovered by cover‐testing, may have also played a part in the development of amblyopia (Lang, 2010). Both cataracts as well as extremely high refractive errors and astigmatism in both eyes were only rarely present.

The special feature of our large cohort study is that we were able to differentiate between different levels of prematurity as well as ROP and its treatment. In our study, amblyopia prevalence was highest in the group treated for ROP as well as the group with a GA ≤28 weeks, which is supported by our further association analyses showing that amblyopia was associated especially with GA ≤28 weeks, while preterm birth after a GA of 29 weeks and higher did not significantly affect amblyopia prevalence or amblyopia in these groups may have been treated successfully. Interestingly, the prevalence of bilateral amblyopia was descriptively highest in the group treated for ROP, while otherwise only rarely present in children born full‐term and children born moderately preterm (GA 29–32 weeks). A GA of ≤28 weeks, placental insufficiency, and ROP treatment were associated with amblyopia, which had also been the case in our previous analyses in adults in the Gutenberg Prematurity Eye Study (GPES) (Fieß, Greven, et al., 2022). An association of placental insufficiency with strabismus has to our knowledge not been described elsewhere; however, an association of a heavier placenta at birth and strabismus has been described previously by Lingham et al. (2020). These findings suggest that conditions affecting placental function could potentially be associated with an increased risk of strabismus. Further research specifically examining the relationship between the factors for placental insufficiency, placental weight, and strabismus is needed to establish a clearer understanding of this potential link (Carlton & Kaltenthaler, 2011).

Amblyopia is primarily characterized by reduced visual acuity, without an organic cause, which in our cohort was observed in association with GA, BW, BW percentile, ROP, ROP‐treatment, and perinatal adverse events. Previous studies have reported increased ophthalmologic disorders and reduced visual function in children born preterm. Dobson et al. (2006) found that in cases of ROP, more severe retinal residuals (such as abnormally straightened temporal retinal vessels, macular heterotopia, retinal fold, or partial/total retinal detachment) were associated with worse visual acuity. Holmstrom & Larsson (2008) who assessed reduced visual acuity of ≥0.1 logMAR in children with a BW of ≤1500 g, found that 25% had visual dysfunction at 10 years of age and that neurological complications, cryotreated ROP, anisometropia, and astigmatism were risk factors, which further supports our findings. Additionally, they found significant associations between visual impairment and treated ROP as well as BW (Holmstrom et al., 2014). The effects of amblyopia, previously summarized in a systematic review by Carlton & Kaltenthaler (2011) in combination with our results, emphasize the large effect that prematurity and especially ROP‐treatment can have on the visual‐related quality of life of these children, such as increased bullying, feelings of isolation and low self‐esteem, to only mention some.

There are several studies that have assessed the influence of factors associated with preterm birth on the development of refractive errors during childhood (Darlow et al., 1997; Fieß, Kolb‐Keerl, Schuster, et al., 2017; Fledelius, 1996; Larsson et al., 2003; O'Connor et al., 2006; Quinn et al., 1998).

In our cohort, the main refractive cause for amblyopia was anisometropia (spherical or cylindrical), while extreme myopia/hyperopia and/or astigmatism as causes were rare. Although anisometropia (spherical, continuous) was descriptively higher in the group with individuals born extremely preterm as well as both ROP and ROP treatment, anisometropia (spherical, continuous) was only associated with a GA ≤28 weeks but not with moderate or late prematurity or ROP and/or ROP treatment. Anisoastigmatism did not show any relevant associations regarding perinatal factors in multivariable analysis in our cohort. It could be hypothesized that anisometropia rather than anisoastigmatism contributed to the amblyopia in our cohort when considered in relation to prematurity.

Participants treated for ROP were significantly more myopic in descriptive analysis compared with the term‐born group (*p* ≤ 0.01) which has also been described in a previous analysis (Fieß, Fauer, et al., 2022). Spherical equivalent was furthermore associated with ROP treatment in age‐ and sex‐adjusted analysis, which is in line with the finding that high refractive error (myopic or hyperopic) was a cause of amblyopia in this group, despite the small sample size. Other studies have described an increase in more extreme hyperopia and myopia cases in participants with ROP treatment compared with term‐born controls (Larsson et al., 2003).

A direct influence of perinatal adverse events on astigmatism and anisoastigmatism specifically has, to our knowledge, not been described in previous research; however, perinatal adverse events have been associated with visual impairment and neurodevelopmental challenges (Rees et al., 2022) as well as with amblyopia (Cao et al., 2023). Perinatal adverse events, as defined in our study (intraventricular haemorrhages, bronchopulmonary dysplasia, and necrotizing enterocolitis) (Institute for Quality Assurance and Transparency in Healthcare (IQTIG), 2023), may contribute to astigmatism and anisoastigmatism through increased oxidative stress (Goulding et al., 2020), which has been described as a factor in corneal development (Nita & Grzybowski, 2016).

Further, amblyopia can be caused by ptosis or cataracts. In our cohort, we did not have any participants with ptosis. The increased size of lens opacifications in children with ROP treatment demonstrates that these lens changes start in early life. Christiansen & Bradford (1995) previously identified a higher rate of lens opacifications and cataracts after early ROP treatment with argon laser. Clinically, this may be significant, as these anatomical changes could contribute to increased refractive error, reduced accommodation amplitude, and potentially a heightened risk of cataract development later in life as indicated by others (Fieß, Fauer, et al., 2022), as well as to amblyopia.

### 4.1 Strengths and limitations

Our study design was limited by being single‐centre and hospital‐based. A portion of potential participants declined participation, contributing to possible selection bias. Some participants were difficult to contact, and individuals with greater morbidity may have been underrepresented, potentially skewing the results towards those with less severe impairments. Additionally, the study included only a small number of participants with advanced stages of ROP, potentially affecting the generalizability of long‐term outcomes. Most participants were Caucasian, limiting the applicability of results to other ethnic groups. Furthermore, factors such as family history of refractive error, cerebral lesions, and cognitive delay could not be fully adjusted for, which may influence the reported outcomes. An additional limitation of this study is that near visual acuity was not examined, potentially overlooking important indicators of visual difficulties in tasks such as reading and other classroom activities that are critical for learning and development in children. Furthermore, our definition of amblyopia was not age‐adjusted and may therefore lead to potential misclassification in younger children, as it does not fully account for normal variations in visual acuity development. We also need to acknowledge the limitation that our approach to amblyopia aetiology does not differentiate between primary organic deprivation from ROP and secondary consequences related to ROP. We were also not able to make any statements regarding the effect of our results on the visual‐related quality of life; however, previous results from a systematic review suggest that prematurity and especially ROP treatment can have large effects on the visual‐related quality of life, such as increased bullying, feelings of isolation, and low self‐esteem (Carlton & Kaltenthaler, 2011). In addition, not all participants in Group 2 underwent a standard ROP screening, leaving a small possibility of undetected postnatal ROP; however, this risk is deemed very low since all children were treated according to the German Retinological Society's guidelines (Clemens et al., 1999; Jandeck et al., 2008).

Despite these limitations, this study stands out for examining one of the largest cohorts of individuals born preterm during childhood, stratified into extreme, moderate, and late preterm birth as well as with and without ROP and/or treatment. This study allows for a valuable, long‐term perspective on prematurity's effects on amblyopia, visual acuity, refractive error, and lens opacification. The comprehensive data gathered from perinatal records enabled a robust analysis of factors influencing these outcomes, with visual assessments conducted by masked investigators, minimizing investigator bias. All examinations followed strict standardized protocols, enhancing the reliability of the findings and providing a clear view of the associations between prematurity, ROP, and long‐term visual outcomes.

## 5 CONCLUSIONS

This study assessed amblyopia and prematurity‐related refractive errors, stratified by different levels of prematurity and the presence and absence of ROP, as well as ROP treatment during childhood. Our findings indicate that extreme prematurity (GA ≤ 28 weeks) and ROP treatment pose a higher risk for amblyopia, while moderate preterm birth (GA ≥ 29 weeks) was not associated with amblyopia. Regarding refractive errors that might affect amblyopia genesis, ROP treatment was associated with a more myopic spherical equivalent, while anisometropia was mainly associated with extreme prematurity but not with ROP or ROP treatment. This may suggest differences in the underlying reasons for amblyopia genesis dependent on the stage of prematurity and ROP and/or its treatment. Comprehensive ophthalmologic follow‐ups remain essential for early intervention, particularly in children with histories of preterm birth and ROP, to manage potential long‐term impacts on vision.

## CONFLICT OF INTEREST STATEMENT

AF, SaG, AH, EM, HE, PL, CK, BS, StG: No financial disclosures. NP receives financial support and grants from Novartis, Ivantis, Santen, Thea, Boehringer Ingelheim Deutschland GmbH & Co. KG, Alcon, and Sanoculis. AKS receives research support from Allergan, Bayer, Heidelberg Engineering, PlusOptix and Novartis.

## Supporting information


Appendix S1.

Figure S2.

## Data Availability

AF had full access to all the data in this study and took responsibility for the integrity of the data and the accuracy of the data analysis. Statistical analyses were performed by AF and SG. The analysis presents clinical data of a cohort. This project constitutes a major scientific effort with high methodological standards and detailed guidelines for analysis and publication to ensure scientific analyses are at the highest level. Therefore, data are not made available for the scientific community outside the established and controlled workflows and algorithms. To meet the general idea of verification and reproducibility of scientific findings, we offer access to data at the local database upon request at any time. Interested researchers make their requests to the coordinating PI (Achim Fieß; achim.fiess@unimedizin‐mainz.de). More detailed contact information is available at the homepages of the UM (http://www.unimedizin‐mainz.de).
