# Supplementary material for: Visual acuity, amblyopia and refractive error in preterm children with and without retinopathy of prematurity – Results from the Gutenberg Prematurity Study Young (GPSY)
Source: Acta Ophthalmol. 2025 May 28;103(7):e472–84. doi: 10.1111/aos.17515 (PMC12531598; doi:10.1111/aos.17515)
Supplement: Supplementary file 1 — Appendix S1. [file AOS-103-e472-s001.docx]

Supplemental Material


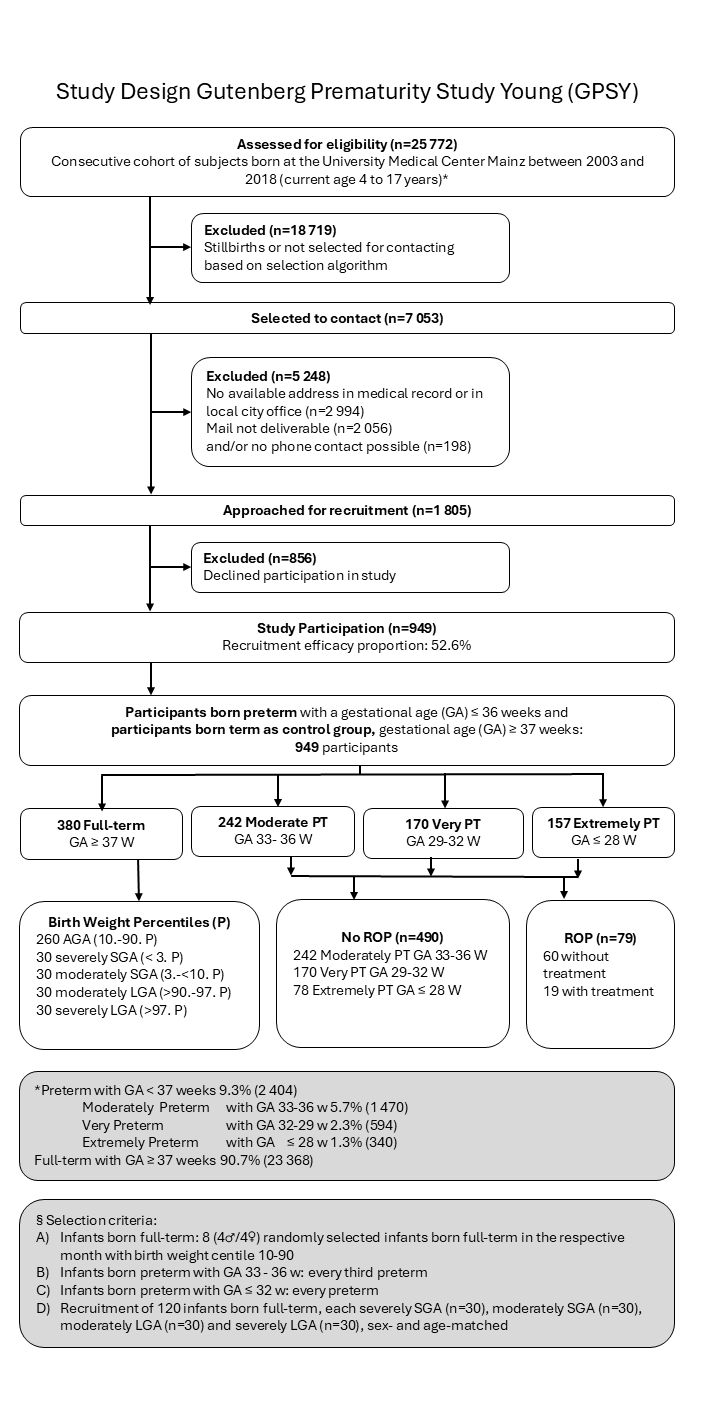
Supplementary Figure S1. Study Design of the Gutenberg Prematurity Study Young (GPSY)

GA – gestational age, ROP – retinopathy of prematurity, AGA – appropriate for GA, SGA - small for GA, LGA – large for GA, PT – preterm, w - weeks

Supplemental Figure 2:

Figure 2. Graphical Display of Refractive Error and Visual Function


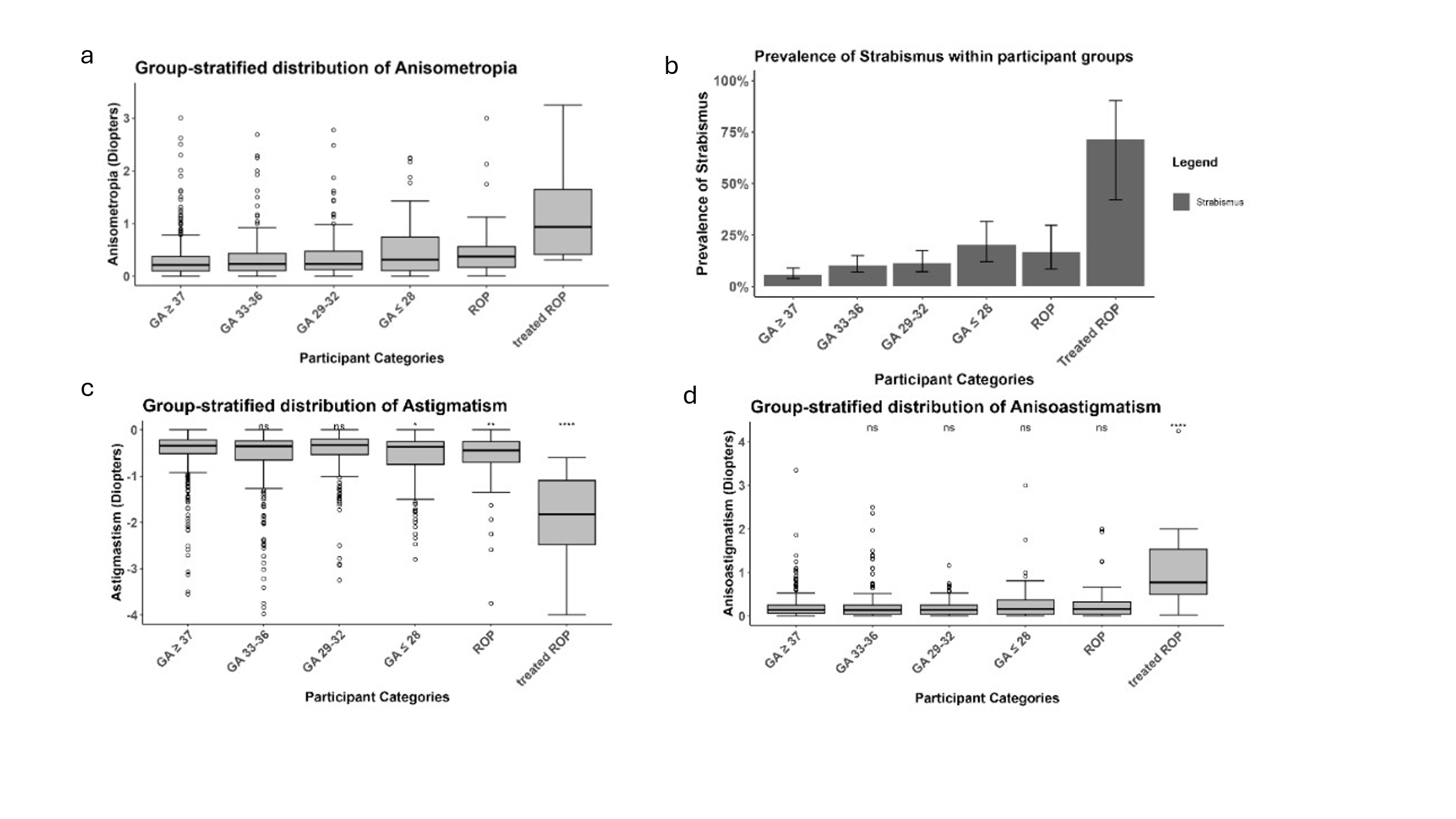


#### GA – Gestational age in weeks, ROP – retinopathy of prematurity

Group-stratified distributions of a) Anisometropia, b) Prevalence of Strabismus, c) Astigmatism, d) Anisoastigmatism, comparisons against term-born group (GA ≥ 37): ns – p >0.05, * - p ≤ 0.05, ** - p ≤ 0.01, *** - p ≤ 0.001, **** - p ≤ 0.0001, Whiskers in b): 95%-Confidence-Interval

Supplemental Material Table S1.

Characteristics of the GPSY Sample (n=949), stratified by study groups

|  | **Group 1**  **GA ≥ 37 wks** | **Group 2**  **GA 33-36 wks**  **No ROP** | **Group 3**  **GA 29-32 wks**  **No ROP** | **Group 4**  **GA ≤ 28 wks**  **No ROP** | **Group 5**  **GA ≤ 32 wks**  **ROP without treatment** | **Group 6**  **GA ≤ 32 wks**  **ROP with treatment** |
| --- | --- | --- | --- | --- | --- | --- |
| Participants (n)/ eyes (n) | 380/760 | 242/484 | 170/340 | 78/156 | 60/114 | 19/35 |
| Gender (female) | 195 (51.3) | 120 (49.6) | 90 (52.9) | 47 (60.3) | 34 (56.7) | 9 (47.4) |
| Age, y (mean (SD)) | 11.04 (3.72) | 11.17 (3.75) | 11.69 (3.93) | 10.79 (3.94) | 11.93 (5.39) | 10.47 (4.30) |
| Gestational age (weeks) (mean (SD)) | 38.98 (1.29) | 34.64 (1.04) | 30.89 (1.05) | 26.17 (1.45) | 26.43 (2.35) | 24.05 (1.08) |
| Birth weight, kg (mean (SD)) | 3.38 (0.64) | 2.33 (0.41) | 1.57 (0.35) | 0.85 (0.25) | 0.93 (0.35) | 0.62 (0.20) |
| Birth weight < 1500g (yes) (%) | 1 (0.3) | 7 (2.9) | 74 (43.5) | 78 (100.0) | 57 (95.0) | 19 (100.0) |
| Birth weight < 1000g (yes) (%) | 0 (0.0) | 0 (0.0) | 10 (5.9) | 58 (74.4) | 40 (66.7) | 18 (94.7) |
| Birth weight percentile (mean (SD)) | 46.24 (33.40) | 33.98 (22.83) | 40.47 (22.37) | 36.79 (26.11) | 42.23 (27.11) | 22.95 (24.26) |
| Birth Weight Percentile Categories |  |  |  |  |  |  |
| - <3. Percentile | 30 (7.9) | 11 (4.5) | 5 (2.9) | 7 (9.0) | 1 (1.7) | 3 (15.8) |
| - 3.-<10. Percentile | 30 (7.9) | 31 (12.8) | 11 (6.5) | 7 (9.0) | 9 (15.0) | 4 (21.1) |
| - 10.-90. Percentile | 260 (68.4) | 197 (81.4) | 154 (90.6) | 62 (79.5) | 50 (83.3) | 12 (63.2) |
| - >90.-97. Percentile | 30 (7.9) | 2 (0.8) | 0 (0.0) | 2 (2.6) | 0 (0.0) | 0 (0.0) |
| - >97. Percentile | 30 (7.9) | 1 (0.4) | 0 (0.0) | 0 (0.0) | 0 (0.0) | 0 (0.0) |
| ROP (yes) (%) | 0 (0.0) | 0 (0.0) | 0 (0.0) | 0 (0.0) | 60 (100.0) | 19 (100.0) |
| ROP-Stage (1/2/3/4/5) OD (eyes) | (0/0/0/0/0) | (0/0/0/0/0) | (0/0/0/0/0) | (0/0/0/0/0) | (27/15/14/0/0) | (0/2/14/2/1) |
| ROP-Stage (1/2/3/4/5) OS (eyes) | (0/0/0/0/0) | (0/0/0/0/0) | (0/0/0/0/0) | (0/0/0/0/0) | (22/19/17/0/0) | (0/1/12/2/1) |
| Perinatal adverse events (yes)* (%) | 0 (0.0) | 1 (0.4) | 7 (4.1) | 32 (41.0) | 26 (43.3) | 18 (94.7) |
| Preeclampsia (yes) (%) | 7 (1.8) | 21 (8.7) | 30 (17.6) | 12 (15.4) | 12 (0.0) | 2 (10.5) |
| Placental insufficiency (yes) (%) | 2 (0.5) | 8 (3.3) | 6 (3.5) | 6 (7.7) | 3 (5.0) | 2 (10.5) |
| HELLP Syndrome (yes) (%) | 0 (0.0) | 8 (3.3) | 20 (11.8) | 6 (7.7) | 2 (3.3) | 1 (5.3) |
| Gestational diabetes (%) | 46 (12.1) | 19 (7.9) | 21 (12.4) | 3 (3.8) | 4 (6.7) | 0 (0.0) |
| Maternal smoking during pregnancy (yes) (%) | 17 (4.5) | 7 (2.9) | 7 (4.1) | 5 (6.4) | 8 (13.3) | 4 (21.1) |
| Breastfeeding (yes) (%) | 322 (84.7) | 183 (75.6) | 119 (70.0) | 53 (67.9) | 28 (46.7) | 2 (10.5) |

GA – Gestational age; ROP – Retinopathy of prematurity; wks – weeks; n – Number; y – years; SD – Standard deviation; g – grams; HELLP – Hemolysis, elevated liver enzymes, low platelet; OD – right eye; OS – left eye; IQR – Interquartile range; D – Diopter;

*Perinatal adverse events were defined as the occurrence of intraventricular hemorrhage (at least grade 3 or parenchymal hemorrhage) and/or occurrence of necrotizing enterocolitis and/or bronchopulmonary dysplasia (+moderate or severe).

Supplemental Material Table S2.

| **Astigmatism (Diopters)** |  |  |  |  |  |  |
| --- | --- | --- | --- | --- | --- | --- |
| Quantile Regression (right eyes) | β_(_τ_50)_ [95%-CI] | p | β_(_τ_50)_ [95%-CI] | p | β_(_τ_50)_ [95%-CI] | p |
| Weeks of prematurity (40 weeks – GA) | -0.01 [-0.012;-0.005] | **<0.001** | -0.004 [-0.008; 0.006] | 0.17 | - | - |
| Birth weight (kg) | 0.03 [0.02;0.05] | **0.03** | * | * | * | * |
| Birth weight percentile | 0.0004 [-0.0001;0.0001] | 0.22 | - | - | - | - |
| ROP (yes) | -0.24 [-0.36; -0.12] | **0.004** | - | - | -0.09 [-0.18; -0.02] | 0.17 |
| ROP treatment (yes) | -1.61 [-2.03; -1.35] | **<0.001** | - | - | -1.42 [-1.89; -1.11] | **<0.001** |
| Perinatal adverse events (yes) | -0.25 [-0.45; -0.13] | **0.004** | -0.22 [-0.34; -0.08] | **0.02** | -0.14 [-0.23; -0.04] | **0.03** |
| Smoking during pregnancy (yes) | -0.03 [-0.21;0.02] | 0.68 | - | - | - | - |
| Preeclampsia (yes) | -0.09 [-0.18;0.01] | 0.15 | - | - | - | - |
| Breastfeeding (yes) | 0.04 [-0.02;0.08] | 0.25 | - | - | - | - |
| Placental insufficiency (yes) | -0.04 [-0.11;0.04] | 0.47 | - | - | - | - |
| **Anisoastigmatism (Diopters)** |  |  |  |  |  |  |
| Quantile Regression | β_(_τ_50)_ [95%-CI] | p | β_(_τ_50)_ [95%-CI] | p | β_(_τ_50)_ [95%-CI] | p |
| Weeks of prematurity (40 weeks – GA) | 0.002  [-0.004;0.004] | 0.22 | - | - | - | - |
| Birth weight (kg) | -0.01  [-0.03; -0.003] | 0.13 | * | * | * | * |
| Birth weight percentile | -0.0006  [-0.001; -0.0002] | 0.07 | - | - | - | - |
| ROP (yes) | 0.10 [0.01;0.14] | **0.01** | - | - | 0.04 [0.002;0.19] | 0.33 |
| ROP treatment (yes) | 0.60 [0.38; 1.43] | 0.11 | - | - | - | - |
| Perinatal adverse events (yes) | 0.09 [0.03;0.14] | **0.007** | - | - | 0.06 [0.02;0.17] | 0.09 |
| Smoking during pregnancy (yes) | -0.01 [-0.07;0.09] | 0.83 | - | - | - | - |
| Preeclampsia (yes) | 0.01 [-0.04;0.09] | 0.85 | - | - | - | - |
| Breastfeeding (yes) | -0.02 [-0.07;0.02] | 0.31 | - | - | - | - |
| Placental insufficiency (yes) | 0.04 [-0.11;0.09] | 0.53 | - | - | - | - |
| **Amblyopia** | univariable |  | model 1 |  | model 2 |  |
| Logistic Regression | OR [95%-CI] | p | OR [95%-CI] | p | OR [95%-CI] | p |
| Weeks of prematurity (40 weeks – GA) | 1.12 [1.06;1.19] | **<0.001** | 1.07 [0.99;1.15] | 0.07 | - | **-** |
| Birth weight (kg) | 0.55 [0.40;0.74] | **<0.001** | * | * | * | * |
| Birth weight percentile | 0.99 [0.98;1.00] | 0.25 | - | - | - | - |
| ROP (yes) | 3.42 [1.60;6.86] | **<0.001** | - | - | 0.93 [0.21;2.77] | 0.90 |
| ROP treatment (yes) | 18.47 [6.62;50.30] | **<0.001** | - | - | 18.31 [4.21;101.4] | **<0.001** |
| Perinatal adverse events (yes) | 4.49 [2.19;8.76] | **<0.001** | 1.89 [0.72;4.97] | 0.20 | - | - |
| Smoking during pregnancy (yes) | 2.76 [1.01;6.46] | **0.03** | 2.12 [0.73;5.26] | 0.13 | - | **-** |
| Preeclampsia (yes) | 0.86 [0.25;2.21] | 0.78 | 0.53 [0.15;1.42] | 0.26 | - | - |
| Breastfeeding (yes) | 0.47 [0.26;0.87] | **0.01** | 0.77 [0.40; 1.51] | 0.43 | - | - |
| Placental insufficiency (yes) | 5.35 [1.87;13.35] | **<0.001** | 3.56 [1.16; 9.59] | **0.02** | 4.74 [1.55; 12.54] | 0.003 |
| **Anisometropia (Diopters)** |  |  |  |  |  |  |
| Quantile Regression | β_(_τ_50)_ [95%-CI] | p | β_(_τ_50)_ [95%-CI] | p | β_(_τ_50)_ [95%-CI] | p |
| Weeks of prematurity (40 weeks – GA) | 0.009  [0.004; 0.01] | **0.002** | 0.007  [0.002; 0.01] | **0.02** | 0.004 [-0.0001; 0.01] | 0.19 |
| Birth weight (kg) | -0.03 [-0.06; -0.01] | **0.01** | * | * | * | * |
| Birth weight percentile | -0.0001 [-0.0008;0.0002] | 0.62 | - | - | - | - |
| ROP (yes) | 0.19 [0.12;0.26] | **<0.001** | - | - | 0.12 [-0.02;0.20] | 0.07 |
| ROP treatment (yes) | 0.64 [0.20;1.51] | **0.03** | - | - | 0.48 [0.002;1.02] | 0.17 |
| Perinatal adverse events (yes) | 0.17 [0.05;0.26] | **0.009** | 0.10 [-0.04;0.2] | 0.14 | - | - |
| Smoking during pregnancy (yes) | 0.0004 [-0.07;0.05] | 0.99 | - | - | - | - |
| Preeclampsia (yes) | 0.04 [0.02;0.17] | 0.52 | - | - | - | - |
| Breastfeeding (yes) | -0.005 [-0.05;0.03] | 0.79 | - | - | - | - |
| Placental insufficiency (yes) | -0.001 [-0.09;0.17] | 0.99 | - | - | - | - |

Univariable – adjusted for sex and age, Model 1: Multivariable model with inclusion of univariable associated parameters with adjustment for age and sex. Model 2: Multivariable model with inclusion of associated parameters of model 1 and additional inclusion of ROP occurrence/ROP treatment. Because of a high collinearity with gestational age the parameter birth weight was not included in the multivariable models. OR - Odds ratio, CI - Confidence interval.
