# Supplementary figures and images for: Visual acuity, amblyopia and refractive error in preterm children with and without retinopathy of prematurity – Results from the Gutenberg Prematurity Study Young (GPSY)
Source: Acta Ophthalmol. 2025 May 28;103(7):e472–84. doi: 10.1111/aos.17515 (PMC12531598; doi:10.1111/aos.17515)

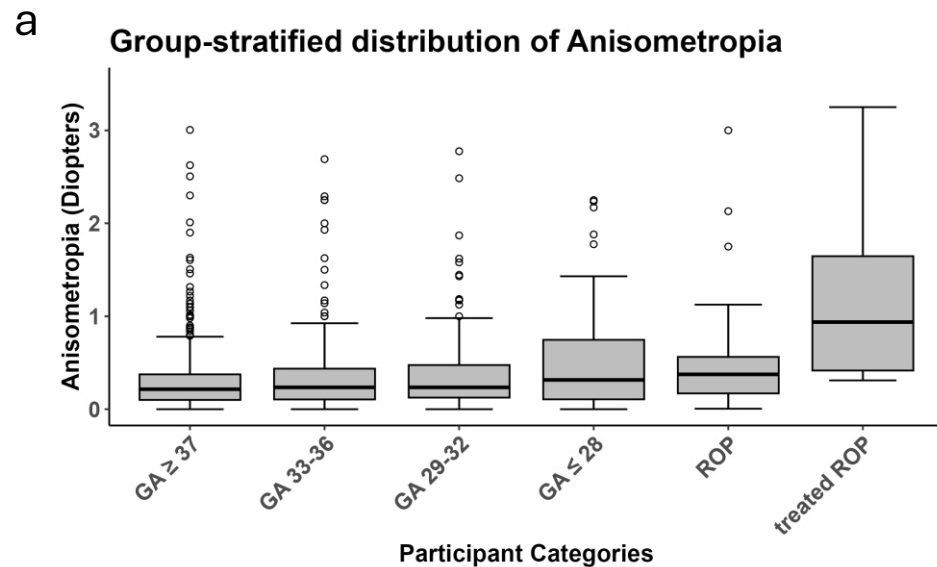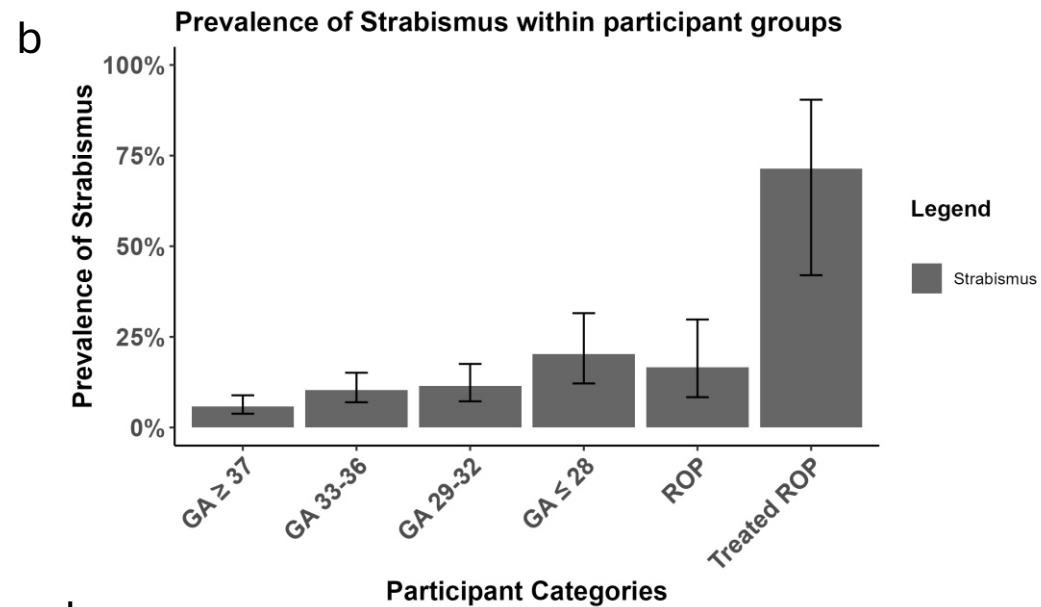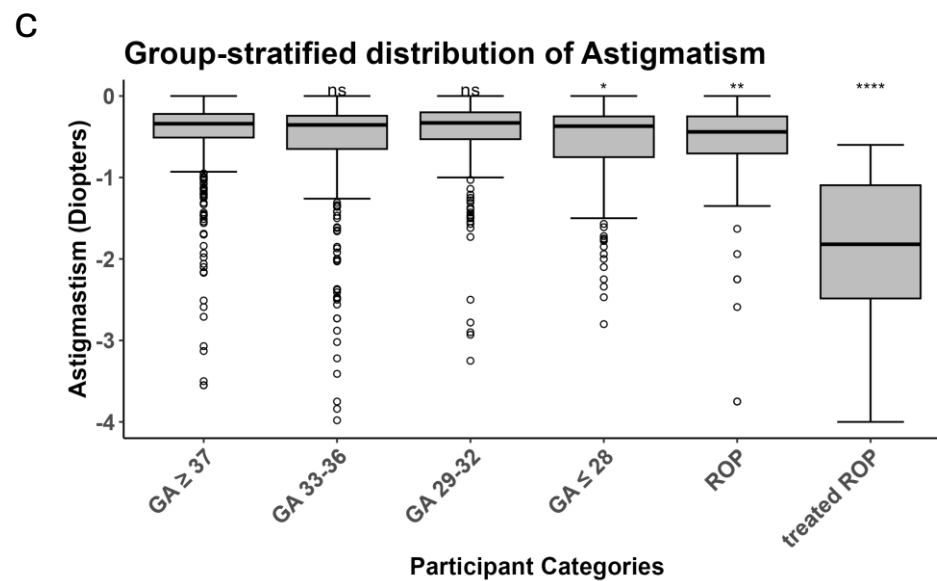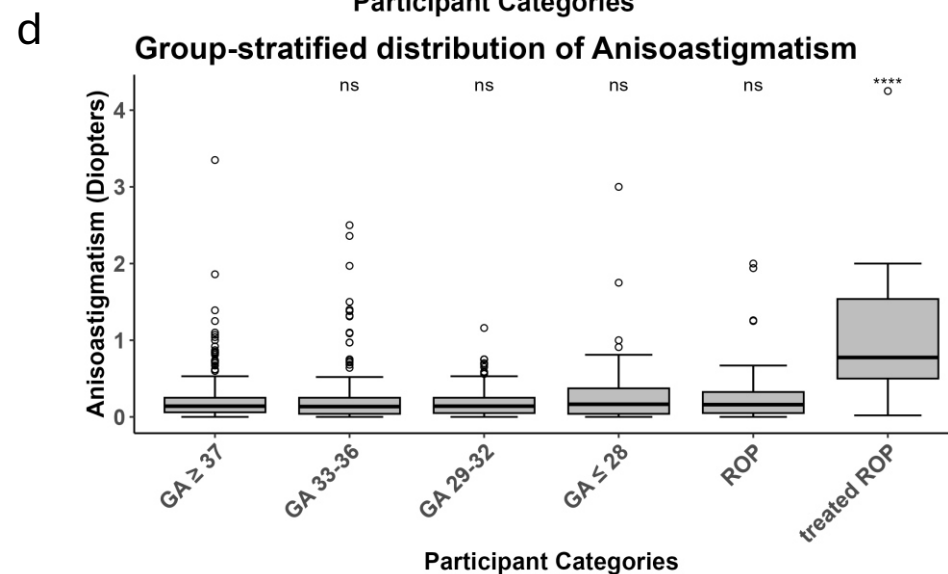

Supplement: Supplementary file 2 — Figure S2. [file AOS-103-e472-s002.pdf]
